# Iron Incorporation and Post-Malaria Anaemia

**DOI:** 10.1371/journal.pone.0002133

**Published:** 2008-05-07

**Authors:** Conor P. Doherty, Sharon E. Cox, Antony J. Fulford, Steven Austin, David C. Hilmers, Steven A. Abrams, Andrew M. Prentice

**Affiliations:** 1 Nutrition Program, Keneba Field Station, Medical Research Council, Fajara, The Gambia; 2 Medical Research Council International Nutrition Group, Nutrition & Public Health Intervention Research Unit, London School of Hygiene & Tropical Medicine, London, United Kingdom; 3 Medical Research Council Human Nutrition Research, Elsie Widdowson Laboratory, Cambridge, United Kingdom; 4 Department of Pediatrics, Baylor College of Medicine and Texas Children's Hospital, Houston, United States of America; University of Oxford, United Kingdom

## Abstract

**Background:**

Iron supplementation is employed to treat post-malarial anaemia in environments where iron deficiency is common. Malaria induces an intense inflammatory reaction that stalls reticulo-endothelial macrophagal iron recycling from haemolysed red blood cells and inhibits oral iron absorption, but the magnitude and duration of these effects are unclear.

**Methodology/Principal Findings:**

We examined the red blood cell incorporation of oral administered stable isotopes of iron and compared incorporation between age matched 18 to 36 months old children with either anaemia post-malaria (n = 37) or presumed iron deficiency anaemia alone (n = 36). All children were supplemented for 30 days with 2 mg/kg elemental iron as liquid iron sulphate and administered ^57^Fe and ^58^Fe on days 1 and 15 of supplementation respectively. ^57^Fe and^58^Fe incorporation were significantly reduced (8% vs. 28%: p<0.001 and 14% vs. 26%: p = 0.045) in the malaria vs. non-malaria groups. There was a significantly greater haemoglobin response in the malaria group at both day 15 (p = 0.001) and 30 (p<0.000) with a regression analysis estimated greater change in haemoglobin of 7.2 g/l (s.e. 2.0) and 10.1 g/l (s.e. 2.5) respectively.

**Conclusion/Significance:**

Post-malaria anaemia is associated with a better haemoglobin recovery despite a significant depressant effect on oral iron incorporation which may indicate that early erythropoetic iron need is met by iron recycling rather than oral iron. Supplemental iron administration is of questionable utility within 2 weeks of clinical malaria in children with mild or moderate anaemia.

## Introduction

In endemic countries malaria produces a flux of iron from the haemolysis of red blood cells in infants and young children many of whom are already iron deficient. The relative contributions of malaria and iron deficiency to post-malaria anaemia are often unclear, however iron supplementation combined with effective anti-malarial therapy is commonly employed and has been shown to be an effective strategy for the management of post-malarial anaemia [1].

Haemolysis, haemoglobin recycling and iron flux are central to the pathophysiology of malaria and post-malarial anaemia. Haemoglobin is recycled predominately through reticulo-endothelial macrophages to the bone marrow to fulfill erythropoetic iron need. Inflammatory insults however inhibit the release of iron from macrophages [2] and malaria can result in a sequestration of iron in reticuloendothelial macrophages [3] inhibiting iron availability to erythroblasts. Inflammation can also result in the inhibition of oral iron absorption [4] but the magnitude and duration of this effect after acute malaria are unclear. Malaria does not cause or exacerbate iron deficiency because iron does not exit the body in significant amounts, and we hypothesized that therapeutic supplementation of iron for post-malarial anaemia may occur in the presence of sequestrated macrophagal iron and impaired oral absorption. It is unclear how much erythropoetic iron need is met from recycled haemoglobin or supplemental iron, for how long the blockade of iron recycling persists, and whether accompanying iron deficiency stimulates absorption in the child with malaria in a similar manner to the child with uncomplicated iron deficiency.

We examined and compared the red cell incorporation of orally administered stable isotopes of iron and the haemoglobin response to 30 days of iron supplementation in children anaemic after malaria and in those with non-malaria anaemia.

## Materials and Methods

In The Gambia 76% of children have moderate (<110 g/l) and 15% severe anaemia (<70 g/l) at the end of the malaria season [5]. Malaria transmission (September to November) mainly occurs during and immediately after the rainy season and is of moderate intensity with 1–10 infective bites per person per year (plasmodium falciparum). The malaria season coincides with the ‘hungry’ season when food stores are low when iron deficiency is more prevalent.

### Subjects and methods

Anaemic children (haemoglobin <110 g/l) were recruited from the MRC Keneba clinic during the malaria season of 2003. Children between 18 and 36 months of age with anaemia associated with uncomplicated malaria were recruited from children who presented with a fever and were found to have a peripheral parasitemia of greater than 500 parasites per microliter on Field's staining of a thick blood film. This was later confirmed with Giemsa staining. These children were treated with a standard 3 day course of chloroquine (10 mg/kg/day) and fansidar (½ tablet if less than 15 kg body weight once) and were recruited on the 4^th^ day after diagnosis when treatment was completed. Employing haemoglobin at the time of diagnosis as a baseline when examining the erythropoetic response after malaria is problematic as haemoglobin can continue to drop during and after malaria treatment [3]. Well anaemic children between 18 and 36 months of age with no evidence of malaria parasites on blood film were recruited from either the immunization and growth monitoring clinic or the general clinic. These children were recruited if anaemic without a history of fever in the last 7 days and no medical record of having had a clinical malarial episode documented or having received anti-malarials during the present malarial season. Children with severe malnutrition (<−3 whz scores away from reference NCHS 50^th^ centile) and severe anaemia (<60 g/l) were excluded from the study. The study physician saw all children on days 1, 15 and 30 and documented any clinical symptoms.

All children had a venous blood sample taken on day1 and day15 and thereafter received ^57^Fe (3.9 mg on day1) and ^58^Fe (1.3 mg on day15) early in the morning after at least a 2 hour supervised fast. The iron doses were drawn up by needle and syringe from their respective solutions. The exact weight (Mettle AM100 scales) of the needle and syringe was detailed before and after introduction of the isotope into 20 ml of orange juice and used to calculate the dose of isotope administered to each child. Thereafter each child was administered a crushed 50 mg vitamin C tablet in an additional 20 ml of orange juice. All children were observed for 2 hours after isotope administration and any spillage or vomiting was recorded. From day 2 all children were supplemented daily by a field-worker with 2 mg/kg elemental iron as liquid iron glycine sulphate (Plesmet Syrup: Universal Products, Preston, UK) until day 30 when a final venous sample was taken. No vitamin C was given with the liquid iron glycine sulphate.

### Laboratory methodology

Haemoglobin (Hb) (Medonic CA 530 haemoglobinometer) and zinc protoporphyrin (ZnPP) (Aviv Biomedical haematofluorometer) were measured in The Gambia within 8 hours of sampling and each sample was assessed for the presence of malaria parasites as described above. Frozen plasma was transported to the UK for the analysis of ferritin (Imx ferritin assay based on Microparticle Enzyme Immunoassay (MEIA) technology, Abbot Laboratories), soluble transferrin receptors (STfR) (R&D systems ELISA), erythropoetin (Epo) (R&D systems Quantikine IVD ELISA) and C reactive protein (CRP)(Dade Dimension particle enhanced turbidimetric immunoassay).

### Stable isotope incorporation methodology

Iron-57 and 58 were converted to an aqueous solution of iron sulphate as previously described [6]. Isotope ratios were measured by thermal ionization magnetic sector mass spectrometry [6], [7]. Ratios were expressed relative to the non-administered isotope iron-56 and corrected for temperature specific differences in fractionation using the ratio of iron-54 to iron-56. Iron isotope ratios were converted to tracer:tracee ratios as described elsewhere [8] and red blood cell incorporation was expressed as percentage of dose administered.

### Ethics and Consent

The clinic of the MRC Keneba field-station has provided free medical care for local villages for over 25 years. Detailed medical records of growth and morbidity are maintained for all children under 3 years of age. The nearest government facility is 20 km away and it is very unusual for a child from the local villages to receive treatment outside of the MRC Keneba clinic. The parents/guardians of children were approached after attendance at the MRC Keneba clinic for either routine monitoring or after emergency treatment for malaria. A field-worker not attached to the clinic team explained the study procedure by reading out an information sheet in Mandinka. Individual informed consent was obtained and confirmed by signature/thumbprint. All children were provided with an insecticide treated bed net on entry to the study if they did not already sleep under one. The study was approved by the MRC/Gambian Government Joint Ethics Committee and the The Institutional Review Board for Baylor College of Medicine and Affiliated Hospitals in Houston, USA.

### Statistics and data analysis

Based on variance data from previous similar studies a sample size of 38 per group was calculated. We aimed for 90% power to demonstrate a significant difference (5%) in isotope incorporation between children with malaria (estimated mean (s.d.) incorporation 20%+/−15%) and children without malaria (estimated mean (s.d.) incorporation 35%+/−25%). Microsoft Access (2000) was used for data recording and Epi-Info (v6.04) for generation of nutritional z scores. All data was dual entered with a validation performed between the two entry records and against the hard copy of the data. Differences between groups were compared using t-tests for quantitative variables (or transformed variables) with approximately normal distributions and chi-squared tests for categorized variables (SPSS 11.5.0). The strength of trends was measured by Spearman correlations. A linear regression model of iron absorption, adjusting for haemoglobin at day1, was used to compare differences between post-malaria anaemia and non-malaria anaemia groups and to estimate the marginal means of log_e_ stable isotope incorporation at day 1 and 15 for the malaria and non-malaria groups. A multilevel model was utilized to assess the effects of whz, age, haemoglobin, CRP and iron sufficiency markers on isotope incorporation independent of day of administration.

## Results

There was no significant difference in age or whz score between children with post-malarial anaemia (n = 37) and those with non-malaria anaemia (n = 36) (Table 1). The malaria group had a significantly lower mean haemoglobin on day1. All children were observed to take their daily supplement for 30 days. Children who either refused to take their stable isotope-labeled iron or who vomited after administration on day 1 (n = 6) or day15 (n = 16) were excluded from analysis of red blood cell incorporation. Six children (malaria group n = 5) who re-presented with fever or history of fever between days 15 and 30 were found to have a positive malaria film and were treated.

**Table 1:** Baseline characteristics (means (S.D.) or geometric means (S.D.)).

	Malaria	Non-malaria anaemia	p
n	37	36	/
Hb (g/l)	83.7 (11.6)	90.6 (12.1)	0.02
Age (years)	2.24 (0.5)	2.29 (0.5)	0.69
Whz	−1.4 (0.86)	−1.3 (0.8)	0.66
*Soluble transferrin receptors (mg/l)	5.9 (1.5)	8.5 (5.8)	<0.000
*Ferritin (ng/ml)	74.1 (74.8)	5.8 (21.5)	<0.000
*Zinc protoporphyrin (µmol/mol hb)	127 (53)	157 (111)	0.06
*Erythropoetin (miu/ml)	59 (205)	32 (62)	0.02

* geometric means.

In the well, non-malaria anaemic group iron deficiency could be defined retrospectively on the basis of cut-offs in at least 2 of 3 iron sufficiency markers i.e. STfR >8.5 mg/l [9], ferritin <12 µg/l unless CRP >10 mg/l then <30, and ZnPP >61 µmol/mol haemoglobin [10]. Of those children on day1 in which all 3 markers were measured (n = 31) in the non-malaria anaemic group 27 fulfilled the criteria for iron deficiency. On days 15, 53 of 70 children with all 3 iron sufficiency markers measured fulfilled the criteria for iron deficiency. The interpretation of baseline iron sufficiency markers in the presence of malaria will be discussed.

**Figure 1:**
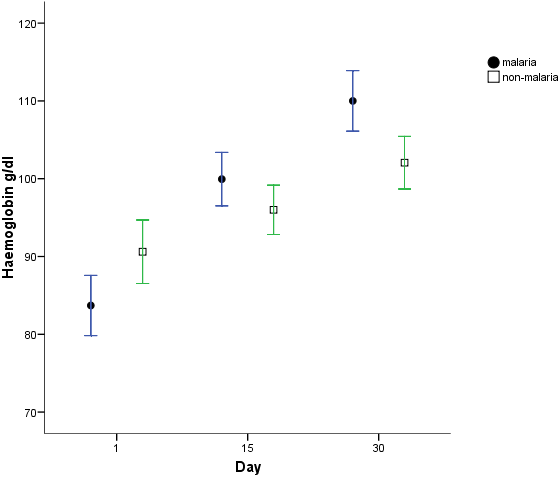
Haemoglobin response (mean & 95% CI).

**Figure 2:**
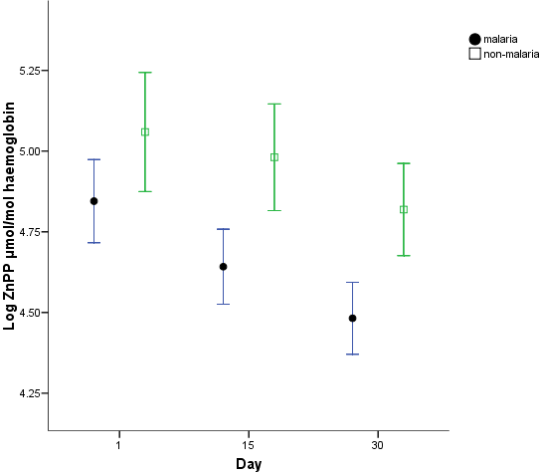
Log_e_ zinc protoporphyrin measurements (mean & 95% CI). Measurements above 4.1 may be indicative of iron deficiency [10].

**Figure 3 pone-0002133-g003:**
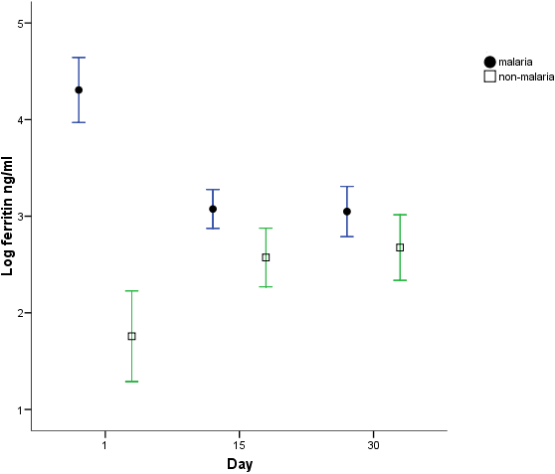
Log_e_ ferritin measurements (mean & 95% CI). Measurements below 2.5 may be indicative of iron deficiency [10].

**Figure 4 pone-0002133-g004:**
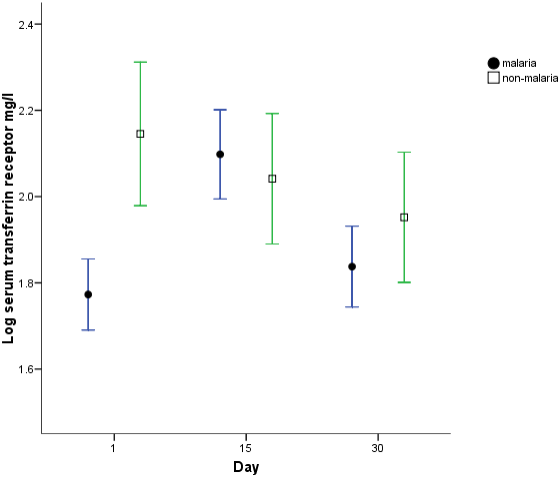
Log_e_ soluble transferrin receptor measurements (mean & 95% CI). Measurements above 2.14 may be indicative of iron deficiency [9].

Analyses of differences in haemoglobin, iron sufficiency marker measurements and stable isotope incorporation were adjusted for differences in day1 haemoglobin between groups. There was a significantly greater haemoglobin response (Figure 1) in the malaria group at both day15 (p = 0.001) and 30 (p<0.001) compared to the non-malaria group with a regression analysis estimated greater change in haemoglobin in the malaria group at day 15 of 7.2 g/l (s.e. 2.0) and at day 30 of 10.1 g/l (s.e. 2.5). ZnPP, ferritin and STfR but not EPO measurements were all significantly different (p<0.001) at day1 in the malaria group (Figures 2–5). ZnPP measurements were high in both groups and subsequently decreased, associated with iron supplementation, but remained significantly higher in the non-malaria group at days 15 (p<0.001) and 30 (p<0.001) (Figure 2). Differences in ferritin measurements decreased between groups by day15 (p = 0.016) and were non-significant by day 30 as measurements normalised. Ferritin measurements increased with supplementation in the non-malaria group and decreased in the malaria group as the inflammatory effect of malaria waned (Figure 3). Differences in STfR measurements became non-significant by day15 as the depressant effect of malaria waned and the measurements reduced towards the normal range in the non-malaria group (Figure 4). Differences widened again by day 30 (p = 0.009) as the malaria group measurements decreased more rapidly. EPO measurements fell in both groups associated with supplementation and haemoglobin response. The fall was more marked in the malaria group by day15 when measurements were significantly different (p = 0.013).

**Figure 5:**
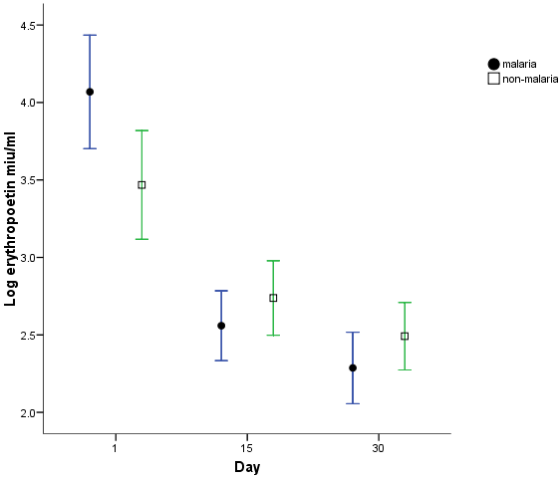
Log_e_ erythropoetin measurements (mean & 95% CI). Measurements above 2.8 are above the normal range (R&D systems).

In a crude analysis, not adjusted for differences in day 1 haemoglobin, red blood cell ^57^Fe incorporation on day 1 was significantly lower in the malaria group (malaria: 8.7% (s.e. 1.17), non-malaria: 26.6% (s.e. 1.15), P<0.001) and remained lower on day15 for ^58^Fe incorportion (malaria:15.5% (s.e.1.26), non-malaria: 24.0% (s.e.1.17), P = 0.116). When adjusted for differences in day 1 haemoglobin both ^57^Fe and ^58^Fe incorporation were significantly reduced between groups (t = −5.4, p<0.000 and t = −2.1, p = 0.045, respectively). The estimated marginal mean of ^57^Fe incorporation in the malaria group on day 1 was 8.3% (95% CI: 6.2–11.3) versus 27.9% (95% CI: 20.6–37.9) in the non malaria anaemic group. On day 15 the estimated marginal mean of ^58^Fe incorporation was 14.4% (95% CI: 9.7–21.1) in the malaria group versus 25.9% (95% CI: 17.5–38.4) in the non-malaria anaemic group. When those children that developed malaria during the study were excluded (n = 6 for 7 malaria episodes of which 5 children were in the post malaria anaemia group) day 15 ^58^Fe incorporation remained lower in the post malaria anaemia group (t = 1.98, p = 0.053).

In the presence of malaria only ferritin (r = −0.44, p = 0.013) and crp (r = −0.43, p = 0.049) correlated with percentage incorporation and not haemoglobin, whz or other iron markers (Table 2). Ferritin and CRP were correlated and indicate the overwhelming effect of malaria-associated inflammation on isotope incorporation. There was no significant difference between ^57^Fe incorporation on day1 and ^58^Fe incorporation on day15 in the non-malaria group suggesting no evident effect of 14 days prior iron supplementation on ^58^Fe incorporation.

**Table 2:** stable isotope incorporation correlations (number of observations).

**Malaria group**						
	Hb	Log ferr	Log ZnPP	Log STfR	Log Epo	Log CRP
Log Isotope incorp.	−0.17(34)	−0.43(32)^1^	−0.02(34)	0.14(32)	0.05(30)	−0.35(32)^1^
Hb		−0.36(35)^1^	−0.45(37)^2^	−0.28(35)	−0.7 (33)^2^	−0.15(35)
Log ferritin			0.24(35)	−0.42(35)^1^	0.6 (33)^2^	0.61(35)^2^
Log ZnPP				0.12(35)	0.4(33)^1^	0.4(35)^1^
Log STfR					0.05(33)	−0.17(35)
Log Epo						0.42(33)^1^
**Combined group**						
	Hb	Log ferr	Log ZnPP	Log STfR	Log Epo	Log CRP
Log Isotope incorp.	−0.14(61)	−0.22(51)	0.24(59)	0.29(59)^1^	0.27(58)^1^	−0.44(54)^2^
Hb		0.09(62)	−0.6(70)^2^	−0.29(70)^1^	−0.66(69)^2^	−0.06(64)
Log ferritin			−0.15(62)	0.03(62)	−0.25(62)^1^	0.25(57)
Log ZnPP				0.54(69)^2^	0.52(68)^2^	−0.05(63)
Log STfR					0.45(69)^2^	−0.08(64)
Log Epo						−0.02(64)

The combined non-malaria group represents data of ^57^Fe absorption on Day 1 and ^58^Fe absorption on Day 15. All correlations expressed as Spearman coefficients.

1 p<0.05.

2 p<0.01.

### Combined group

Data from days 1 and 15 from subjects with non-malarial anaemia was combined to enhance the power to examine influences on iron stable isotope incorporation other than malaria (Table 2). CRP was negatively correlated (r = −0.44, p = 0.001) whilst STfR (r = 0.29, p = 0.028), EPO (r = 0.27, P = 0.042) and WHZ (r = 0.42, P = 0.001) were positively correlated. Neither ferritin nor haemoglobin correlated significantly with isotope incorporation. EPO was negatively correlated with Hb (r = −0.60, P<0.001) and positively correlated with both ZnPP (r = 0.52, p<0.001) and STfR (r = 0.45, p<0.001). Whz (range –2.6 to 0.8 whz scores) was significantly positively correlated with iron incorporation but was not correlated with any of the iron sufficiency markers or CRP. In a multilevel regression model controlling for day of isotope administration only whz (coeff 1.1, p<0.001), CRP (coeff −0.69, p = 0.001) and ferritin (coeff –0.65, p = 0.020) were independently associated with iron absorption.

Increasing malnutrition was associated with less isotope incorporation per kg bodyweight. There was a significant difference in isotope incorporation between those with a CRP measurement greater than or less than 10 mg/l (mean (s.d.) 16 (11.5) vs 34.7 (17.7), p<0.001) and between those with a whz score greater than or less than −1 whz (43.2 (17.2) vs 26.6 (17.8), p = 0.001) (Table 3).

**Table 3:** Effects of inflammation and whz score on % stable isotope incorporation (mean (SD) and N) in the combined ^57^Fe and ^58^Fe group.

	CRP<10 mg/l	CRP>10 mg/l	
Whz >−1	42.6 (15.1) n = 16	24.1 (8.2) n = 2	p = 0.12
Whz <−1	30.1 (17.7) n = 27	14.3 (11.7) n = 15	p = 0.02
	p = 0.02	p = 0.3	

## Discussion

A standard clinical response to severe post-malarial anaemia is to supplement with iron and it is a commonly employed therapeutic strategy in sub-Saharan Africa where endemic iron deficiency often co-exists. This study was designed to assess the effect of malaria on the red blood cell incorporation of iron supplements given to treat post-malarial anaemia. As this study is one of the largest iron stable isotope incorporation studies carried out it also allows us to look at the factors affecting iron incorporation in rural African children.

We have demonstrated a significantly greater haemoglobin recovery in those children with post-malarial anaemia despite an initially reduced capacity to incorporate oral supplemental iron. The reduced incorporation of the oral stable isotope of iron was most marked at day 1 when the children had completed therapy for malaria and were clinically well and apyrexial. By day 15 the incorporation had improved and there was only a marginally significant difference from the non-malaria group. The anaemia of malaria results from the haemolysis of both infected and uninfected red blood cells, an inflammatory mediated effect on iron redistribution to reticulo-endothelial macrophages, and an impaired bone marrow response [11]. Pre-existing iron deficiency may also influence recovery from post-malaria anaemia. In our study the inhibitory effect of acute malaria was short-lived and the erythropoetic response was greater than that of iron deficient children given supplemental iron.

Acute clinical malaria has been associated with a reduction in STfR measurement [12] and a rapidly reversible suppression of bone marrow response to EPO [13]. We found significantly reduced levels of STfR measurements in the malaria group indicating reduced erythropoesis, which increased by day 15 to those levels of the non-malarious group and were reflected in the haemoglobin response. Erythropoetin measurements were high and not significantly different between groups on day 1 and reduced by day 15. During the first 15 days of supplementation a decrease in EPO measurement in the non-malaria group was associated with a decrease in STfR measurement whilst in the malaria group a significantly greater drop in EPO measurement was associated with an increase in STfR measurements. This may indicate that iron flux through the reticuloendothelial macrophages and not erythropoetin limits initial erythropoesis in malaria-associated anaemia. Recycling of senescent red blood cells normally makes up 90% of erythropoetic iron need with dietary iron absorption contributing 10% [14]. We found in post-malaria anaemia that incorporation of supplemental iron was significantly reduced initially, which may indicate that initial erythropoetic iron requirements were met through liberation of reticulo-endothelial iron rather than oral supplemental iron. Comparing these erythropoetic responses to a third group of anaemic children post malaria who were not offered oral iron supplementation would best have tested this, however it was deemed unethical not to supplement moderately anaemic children in an environment of endemic iron deficiency. In the absence of this comparator group we cannot say that supplemental iron had no benefit in the early recovery phase from post malarial anaemia.

It is not possible from this data to determine whether pre-existing iron sufficiency differed between the two groups at baseline as malaria (or the inflammation associated with it) directly affects both STfR [15] and Ferritin [16]. ZnPP has been reported to increase with inflammation [17] as well as iron deficiency however measurements were non-significantly lower in the malaria group in this study. Iron deficiency and malaria are the predominate causes of anaemia in this population. Iron deficiency was present in 87% of the non-malaria group on day 1 and even after 14 days of supplementation was present in 76% of all the children at day 15.

Iron absorption is very difficult to measure in the field and red cell incorporation offers an approximate guide to absorption as well as flux of iron to erythropoetic cells. We employed this method in children with malaria as the best available tool to assess the utility of early iron supplementation in the presence of inflammation and iron sequestration. Stable isotope iron incorporation was reduced initially after malaria and the block most likely occurred during absorption through the small intestinal mucosa. This may have been either a cytokine/hepcidin mediated effect and/or possibly resulted from iron loading of the reticuloendothelial macrophages due to malaria. Il-1 and IL-6 regulate hepcidin transcription in mouse models [18] and human iron overload results in increased urinary excretion of hepatocyte-produced hepcidin [19] though the mechanism of iron sensing and the cell involved remains unclear [20]. ^57^Fe incorporation in post-malaria anaemia did not correlate with haemoglobin or iron sufficiency markers except ferritin. Ferritin was most likely a marker of the overwhelming influence of malaria-associated inflammation on iron incorporation as ferritin is an acute phase reactant.

Examining the combined ^57^Fe and ^58^Fe incorporation in the non-malaria group allows for the assessment of influences on iron incorporation in this population other than malaria. Incorporation was variable and only inflammation, whz score and ferritin were independently associated and not age or haemoglobin. We excluded severely malnourished children (>−3 whz scores) from this study but still found that weight-for-height z-score was positively correlated with iron incorporation and this effect was independent of inflammation. This finding is important as it suggests that poorer whz score is associated with a declining ability to incorporate supplemental iron. Previous smaller studies [21] [22] that have tried to address this issue in severely malnourished children have produced conflicting results often confounded by the high levels of infection/inflammation in hospitalized subjects. Supplemental iron given to severely malnourished children early in rehabilitation is potentially hazardous and not recommended [23]. Our finding of a decreased capacity to incorporate iron in less severely malnourished children might represent a protective mechanism that is further enhanced by inflammation.

Iron deficiency is common in areas of malaria endemicity and supplementation of young children improves iron status [24]. We specifically examined the effect of an acute clinical malaria episode on supplemental iron incorporation and compared the erythropoetic response to supplemental oral iron of anaemic children post-malaria with iron-deficient anaemic children. We demonstrated an acute depressant effect on iron flux which dissipates rapidly allowing for an impressive haemoglobin increase despite reduced supplemental iron incorporation. From this study supplemental iron would appear to have had a questionable effect in the immediate aftermath of malaria when erythropoetic iron need can only have been met by the release of sequestrated iron. Malaria results in iron sequestration and reduced iron absorption which may represent a deliberate mechanism to control iron availability. Malaria induced haemolysis causes free non-transferrin-bound iron and free haemoglobin to be released which can cause oxidative stress [25], [26] and adding supplemental iron to this milieu may not improve erythroblastic iron supply.

Distinguishing iron deficiency from iron seqestration immediately after clinical malaria is very difficult even with access to laboratory markers of iron status and erythropoesis and is a common diagnostic dilemma faced by doctors and nurses throughout malaria endemic areas. Supplemental iron is important to children with severe post-malaria anaemia if there is a likelihood of pre-existing iron deficiency. However we have shown that the utility of supplemental iron given in the first two weeks after a malarial episode to childen with mild/moderate anaemia is questionable irrespective of pre-existing iron deficiency. It is likely that the liberation of sequestered iron is a major source for erythropoesis in the early stages of recovery from malaria induced anaemia and re-assessing haemoglobin and/or iron status two weeks after a malaria episode may well better indicate the need for supplemental iron at a time when it is more likely to be incorporated.
